# Simultaneous paraneoplastic cerebellar degeneration, Lambert-Eaton syndrome and neuropathy associated with AGNA/anti-SOX1 and VGCC antibodies

**DOI:** 10.1186/s42466-021-00129-w

**Published:** 2021-05-24

**Authors:** Jonas Feldheim, Cornelius Deuschl, Martin Glas, Christoph Kleinschnitz, Tim Hagenacker

**Affiliations:** 1 Department of Neurology, University Hospital Essen, Essen, Germany; 2 Center for Translational Neuro- and Behavioral Sciences, University Hospital Essen, Essen, Germany; 3 Department of Neurology, Division of Clinical Neurooncology, University Hospital, Essen, Germany; 4 Tumorbiology Laboratory, Department of Neurosurgery, University of Würzburg, Würzburg, Germany; 5 Institute for Diagnostic and Interventional Radiology and Neuroradiology, University Hospital Essen, Essen, Germany

## Abstract

Anti-glial nuclear antibody (AGNA) is an onconeuroal antibody targeting the nuclei of Bergmann glia in the cerebellum and Anti-SRY-related HMG-box 1 (SOX1). It is highly specific for small cell lung cancer (SCLC) and correlates to the appearance of paraneoplastic neurological syndromes such as Lambert-Eaton myasthenic syndrome (pLEMS) and paraneoplastic cerebellar degeneration (PCD) amongst others. Herein, we present a SCLC patient with rapidly progressive PCD, LEMS and axonal polyneuropathy associated with AGNA/SOX1-antibodies, successfully treated with plasma-exchange (PLEX).

## Case report

A 54y-old male patient first complained a proximal, progressive, painless muscular weakness, dizziness and dysphagia. Two months after symptom onset, the patient was diagnosed with pLEMS due to SCLC. By then the symptoms had further progressed including dysarthria, dyspnea and the inability to independently stand-up or climb the stairs. Repetitive stimulation of the peroneal nerve revealed a decrement and increment. Lumbar puncture proved a mild pleocytosis (5 cells/μl, 39 mg/dl proteins, 75 mg/dl glucose, 14 mg/dl lactate). No paraneoplastic antibodies were detected in the serum or CSF. However, antibodies against voltage-gated calcium channels (VGCC) of the P/Q-type could be detected in the serum. SCLC was treated with etoposide/cisplatin, local radiotherapy and prophylactic whole-brain radiation. Both SCLC and pLEMS (treated by 200 mg pyridostigmine per day) responded well and reached a state of stable remission. There was no oncological therapy applied afterwards.

Nine months later, the patient developed rapidly-progressing symptoms such as unsteady gate, vertigo and weakness of the upper limb. The symptoms were initially attributed to the pLEMS but retrospectively indicated the onset of PCD. Therapy with IV immunoglobulins (160 g over 4 days) and 3,4-diaminopyridine (30 mg/day) significantly increased the muscle weakness. Pyridostigmine was administered at a dose of 270 mg/day. VGCC (570 pmol/l) and SOX1-antibodies could be detected in the blood serum. Lumbar puncture was not performed.

**Fig. 1:**
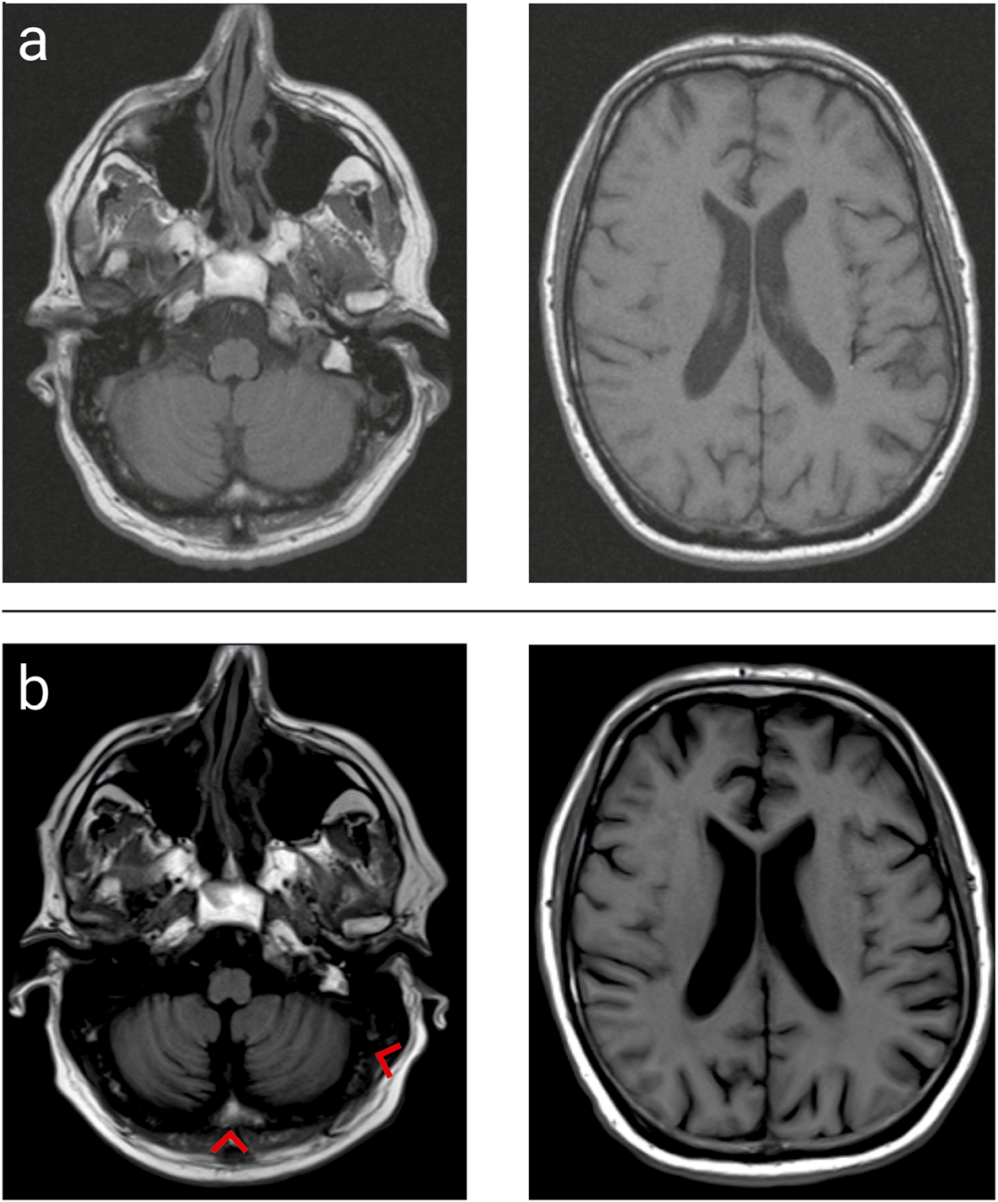
Magnet Resonance Imaging shows progressive atrophy of the cerebellum (indicated by the red arrows) on T1-weighted Turbo spin echo imaging at 10 months (**a**) and 16 months (**b**) after the onset of symptoms, indicative of progressive neurodegeneration. Figure designed with Corel Draw × 8 (Corel Corporation, Ottawa, Canada)

Seven months later, the patient presented at our department for the first time. Ataxia had further progressed. The patient was unable to stand, struggled with eating/drinking and severe dysarthria. An MRI of the brain showed cerebellar atrophy (Fig. 1). Clinical examination revealed a new bilateral paresis (MRC 4/5) of the upper limb, which correlated with axonal motor neuropathy. We verified the pLEMS, which was not detectable in clinical examination, by electrophysiology and occurrence of VGCC autoantibodies in the serum (660 pmol/l). In the CSF, we detected four leucocytes/μl, 53 mg/dl of proteins, 2,8 mmol/l lactate and 90 mg/dl glucose. Increased titers of AGNA (serum 1:320; CSF 1:3.2) were found that were reactive against SOX1 in serum and CSF, as a potential common cause for the PCD, pLEMS and neuropathy.

Treatment with methylprednisolone (cumulative dose of 5 g applied intravenously) had no apparent effect. After diagnosis, the patient was treated with five courses of PLEX. The score in the scale for the rating and assessment of ataxia (SARA) improved from 33 to 25 and (4 weeks later) to 23. The patient re-obtained the ability to sit independently and stand with support. Throughout, the patient continued with physiotherapy that had been started multiple months prior. During his stay at our institution, we registered our patient for a positron emission tomography in combination with a computed tomography (PET-CT) that occurred 4 weeks later. Interestingly, PET-CT revealed a suspected metastasis in the adrenal gland. After the diagnosis of the SCLC, the patient had received regular oncological follow-ups, including CT scans (the last 7 months before the PET-CT), without any new pathological finding.

## Discussion

AGNA is highly specific for SCLC, recognizes SOX1 and is associated in up to 40% with pLEMS that is caused by VGCC antibodies in the blood [1]. However, VGCC autoantibodies that are known to cause pLEMS might play an additional role in this case as they are associated with cerebellar ataxia when present in the CSF [2] and might be a direct effector leading to PCD.

Almost 20% of patients with SOX1-antibodies suffer from PCD, and about 8% develop axonal neuropathy [3]. While the cerebellar degeneration of our patient can be attributed to PCD, the cause of neuropathy may not be conclusively solved. Cisplatin is known to lead to sensory neuropathy; however, an axonal motor neuropathy without the involvement of sensory fibres would be unlikely. Yet, SOX1 antibodies are associated with an axonal neuropathy that may affect motoric nerves [3, 4]. Further investigations revealed no other apparent cause of neuropathy.

PCD severely affected the quality of life due to its rapid progress [5]. Quick and aggressive therapy is vital to prevent irreversible tissue damage. However, the success of immunotherapy is often limited. While autoantibodies to synaptic receptors have been reported as direct effectors of paraneoplastic syndromes, autoantibodies against intracellular antigens, such as SOX1, are believed to be an epiphenomenon [6, 7]. Apoptosis of cancer cells leads to the presentation of cancer-derived antigens to CD4+ helper T cells that stimulate antigen-specific B cells into plasma cells producing the onconeuroal antibodies and activate CD8+ cytotoxic T-lymphocytes which are thought to be responsible for the neurological dysfunction [6, 7]. Therefore, we hypothesized that PLEX might prove an additional effect. In our case, PLEX achieved a good treatment response. Unfortunately, neurodegeneration had already progressed, probably preventing further improvement. Moreover, the rapid progress might have been triggered by the newly diagnosed metastasis in the adrenal gland. However, this offers another therapeutical approach as successful treatment of the metastasis might also improve the symptoms of PCD.

## Conclusion

Delayed onset of PCD should be considered in SCLC patients with pLEMS, and patients should be tested for AGNA/SOX1-antibodies. A rapid progress might be caused by tumor metastasis and PET-CT can assist in diagnosis. If VGCC autoantibodies are present, patients might benefit from PLEX.

## Data Availability

All data generated or analysed during this study are included in this published article.
